# Proteasome-dependent degradation of intracellular carbamylated proteins

**DOI:** 10.18632/aging.102002

**Published:** 2019-06-06

**Authors:** Aurore Desmons, Anaïs Okwieka, Manon Doué, Laëtitia Gorisse, Vincent Vuiblet, Christine Pietrement, Philippe Gillery, Stéphane Jaisson

**Affiliations:** 1 Laboratory of Biochemistry and Molecular Biology, CNRS/URCA UMR N° 7369 MEDyC, Faculty of Medicine, University of Reims Champagne-Ardenne, Reims, France; 2 Laboratory of Pediatric Biology and Research, University Hospital of Reims, Reims, France; 3 Laboratory of Biopathology, University Hospital of Reims, Reims, France; 4 Department of Pediatrics (Nephrology unit), University Hospital of Reims, Reims, France; 5 Present address: Department of Laboratory Medicine, National Institutes of Health, Bethesda, MD 20892, USA

**Keywords:** carbamylation, cell aging, homocitrulline, nonenzymatic post-translational modifications, proteasome, proteostasis

## Abstract

Carbamylation, which corresponds to the binding of isocyanic acid to the amino groups of proteins, is a nonenzymatic post-translational modification responsible for alterations of protein structural and functional properties. Tissue accumulation of carbamylation-derived products and their role in pathological processes such as atherosclerosis or chronic renal failure have been previously documented. However, few studies have focused on the carbamylation of intracellular proteins and their subsequent role in cellular aging. This study aimed to determine the extent of intracellular protein carbamylation, its impact on cell functions and the ability of cells to degrade these modified proteins. Fibroblasts were incubated with cyanate or urea and the carbamylation level was evaluated by immunostaining and homocitrulline quantification. The results showed that carbamylated proteins accumulated intracellularly and that all proteins were susceptible. The presence of intracellular carbamylated proteins did not modify cell proliferation or type I collagen synthesis nor did it induce cell senescence, but it significantly decreased cell motility. Fibroblasts were able to degrade carbamylated proteins through the ubiquitin-proteasome system. In conclusion, intracellular proteins are susceptible to carbamylation but their accumulation does not seem to deeply affect cell function, owing largely to their elimination by the ubiquitin-proteasome system.

## Introduction

Alterations of protein homeostasis ("proteostasis") are among the most important mechanisms responsible for cellular aging [1,2], since cells have to maintain a sufficient proportion of unmodified and functional proteins to assure their physiological roles. However, the systems responsible for the control of proteostasis (*e.g.* chaperones, proteasome, repair enzymes) may become overwhelmed, especially in the event of overproduction of damaged intracellular proteins [3]. Indeed, intracellular proteins are subjected to many nonenzymatic post-translational modifications (NEPTMs) which progressively alter their properties [4]. These modified proteins may be degraded by proteolytic systems, including proteasome, but highly modified proteins may become resistant to proteolysis and accumulate inside cells generating aggregates composed of oxidized and covalently-linked modified proteins also called "lipofuscin" [5,6].

Among the NEPTMs, oxidation and glycation are probably the most studied and it has already been demonstrated that highly oxidized or glycated intracellular proteins may participate in cellular aging. For example, glycated proteins are responsible for the impairment of proteasome activity which further increases their intracellular accumulation [7,8]. Thus, the inability of cells to degrade glycated proteins leads to disruption of proteostasis which constitutes a molecular basis for the development of various age-related or chronic diseases. Indeed, proteostasis impairment in beta cells has been clearly identified as a key process in the progression of type 2 diabetes [9,10]. However, whereas many studies have been devoted to the involvement of oxidized and glycated intracellular proteins in cellular aging, fewer data are available concerning another widespread NEPTM, carbamylation. This reaction corresponds to the nonenzymatic binding of isocyanic acid to functional groups of proteins (especially amino groups), which leads to the formation of carbamylation derived-products (CDPs), the most characteristic being homocitrulline (*i.e.* ε-carbamyl-lysine, HCit) [11]. In humans, isocyanic acid is mainly derived from the spontaneous dissociation of urea into cyanate and ammonia, but may also originate from the myeloperoxidase-catalyzed transformation of thiocyanate.

Like glycation, carbamylation of proteins alters their properties and has been implicated in various pathological contexts such as chronic kidney disease, atherosclerosis or rheumatoid arthritis [12–14]. Our group has recently identified protein carbamylation as a hallmark of tissue aging, characterized by the time-dependent accumulation of carbamylated matrix proteins in skin [15]. However, to our knowledge, no study has yet determined whether carbamylation might play a role in cellular aging.

Evidence for the presence of carbamylated proteins within cells has been presented by Claxton *et al*., who used a qualitative proteomic approach and demonstrated that almost all intracellular proteins are subject to carbamylation [16]. However, their study did not provide any quantitative estimation of the extent of protein carbamylation and was performed using tissue extracts from renal inner medulla, which is exposed *in vivo* to very high concentrations of urea and cannot therefore be considered representative of the general situation.

Thus, the aims of the present study were: (i) to evaluate how carbamylated proteins accumulate inside cells using a quantitative approach, (ii) to analyze the intracellular localization of carbamylated proteins, and (iii) to determine how cells are able to manage these modified proteins. To this end, dermal fibroblasts were incubated with urea or cyanate as carbamylating agents and protein carbamylation assessed either by HCit quantification by liquid chromatography coupled to tandem mass spectrometry (LCMS/MS) or by immunostaining. The role of proteasome in the degradation of intracellular carbamylated proteins was also investigated.

## RESULTS

### Evidence for the presence of carbamylated proteins inside human skin cells

**Figure 1:**
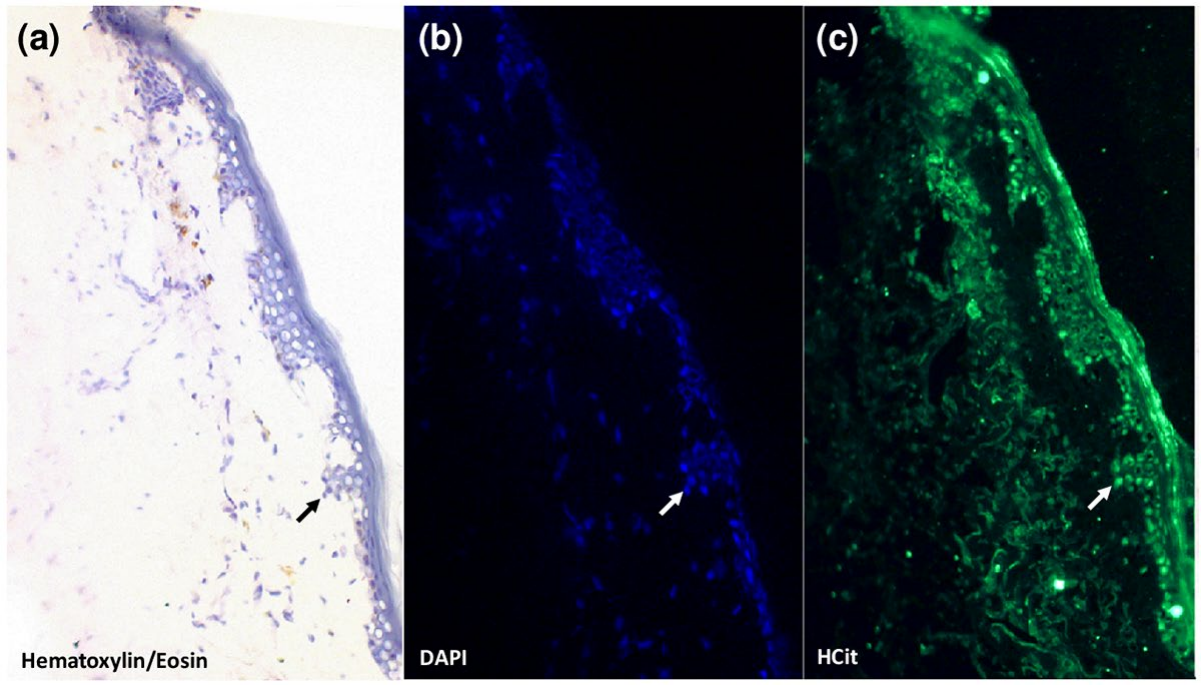
**Evidence for the presence of carbamylated proteins inside human skin cells.** An anti-HCit immunolabelling was realized in a skin section from a 77-year old man counter-stained with DAPI and hematoxylin-eosin. Epidermal area was screened by light microscopy thanks to hematoxylin-eosin counterstaining (**a**). Nuclei were highlighted by DAPI staining (**b**, blue) permitting confirmation of the presence of HCit inside skin cells by immunofluorescence analysis (**c**, green). An example of a cell exhibiting a positive intracellular labelling for HCit is indicated by arrows. Observations were realized at 10x magnification.

Our previous studies have shown that skin HCit content increases with age in different species and that this accumulation is partly due to carbamylation of matrix proteins such as collagen and elastin [15]. In order to determine whether CDPs may also be formed intracellularly, immunohistolabelling analysis of HCit in human skin sections was performed (Fig. 1). This labelling showed the presence of HCit inside cells, providing evidence of intracellular carbamylated proteins.

### Incubations with urea or cyanate lead to an increase of intracellular protein carbamylation level

**Figure 2:**
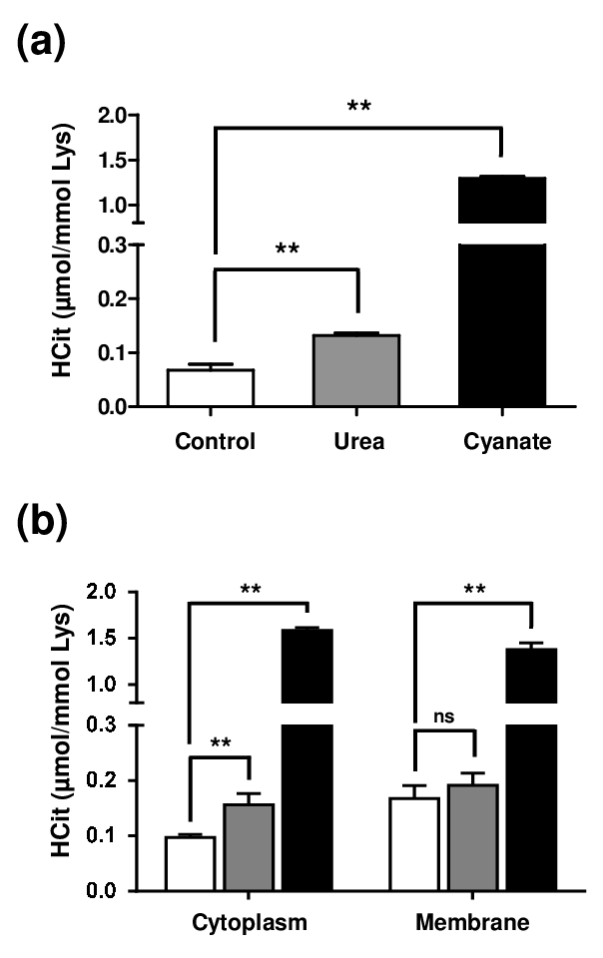
**Intracellular accumulation of carbamylated proteins after long-term incubations with urea or cyanate.** Confluent fibroblasts were incubated for 4 weeks at 37°C with DMEM + 0.5% (v/v) FBS without (control conditions, open bars) or with 20 mmol/L urea (grey bars) or 0.5 mmol/L cyanate (black bars). HCit content was determined by LC-MS/MS in total cell extracts (**a**) and in cytoplasmic and membrane fractions (**b**). The data are presented as means ± SEM (n=6) compared using the Mann-Whitney U test (ns: non significant, **: p<0.01).

In order to evaluate the level of protein carbamylation inside cells, fibroblasts were incubated for 4 weeks with 20 mmol/L urea or 0.5 mmol/L sodium cyanate. Firstly, the results showed a basal carbamylation level of intracellular proteins, since HCit was detectable even without incubation with a carbamylating agent (Fig. 2a). Basal concentrations (0.07±0.01 µmol/mol Lys) were 2-fold and 18-fold increased when fibroblasts were incubated with urea (0.13±0.01 µmol/mol Lys, p<0.01) and with cyanate (1.30±0.02 µmol/mol Lys, p<0.01), respectively.

As results obtained from total cell extracts could be explained only by the carbamylation of membrane proteins exposed to the extracellular medium, another experiment was performed in which membrane and cytosolic fractions were separated. Similar carbamylation levels were found in both membrane and cytoplasmic protein fractions (Fig. 2b) and the same trends as in total extracts were observed in both fractions when the different conditions were compared. In membrane proteins, no significant difference was found between control and urea-incubated cells (0.17±0.02 µmol/mol Lys *vs* 0.19±0.01 µmol/mol Lys) whereas the carbamylation level was significantly increased when cyanate was used as carbamylating agent (1.38±0.03 µmol/mol Lys, p<0.01). In cytoplasmic proteins, HCit content was 0.10±0.01 µmol/mol Lys in basal conditions and reached 0.16±0.02 µmol/mol Lys after incubation with urea and 1.58±0.01 µmol/mol Lys when incubated with cyanate. These results showed that urea as well as cyanate are able to enter cells and induce a significant level of protein carbamylation.

### Actin cytoskeleton and nuclear proteins constitute preferential targets for carbamylation

**Figure 3:**
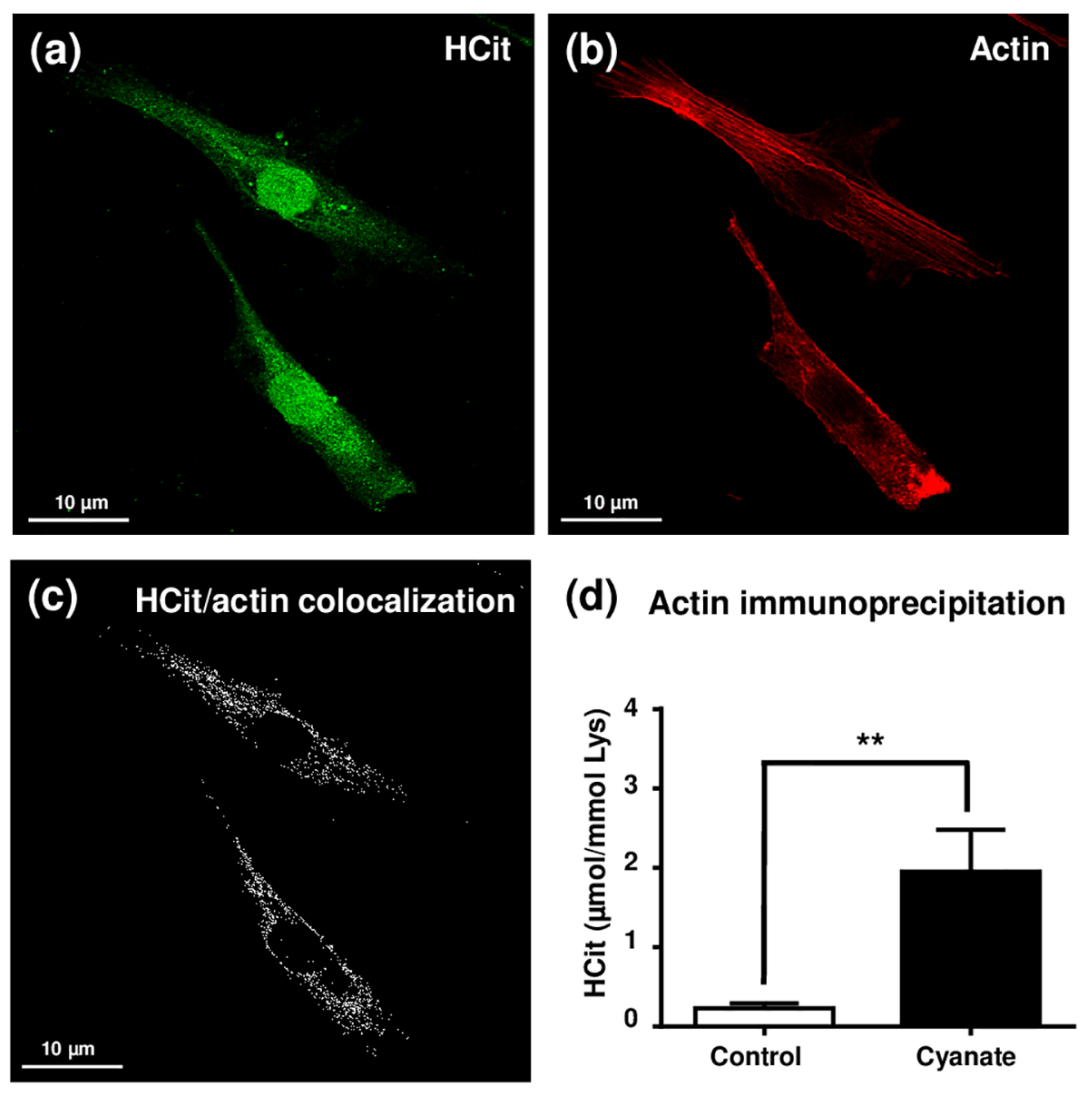
**Localization of intracellular carbamylated proteins.** Fibroblasts were seeded in chambered coverglass system and incubated for 5 days with DMEM containing 0.5% (v/v) FBS and 5 mmol/L cyanate. At the end of incubation, cells were fixed with 4% (v/v) paraformaldehyde and permeabilized with 0.25% (v/v) Triton X-100 before immunolabelling of carbamylated proteins using an anti-HCit polyclonal antibody (**a**). Cells were also labelled using ActinRed 555 ReadyProbes® in order to identify actin fibers (**b**). Colocalization points between HCit and actin labelling were identified using ImageJ software (**c**). In a second set of experiments, fibroblasts were incubated in the same conditions without (control) or with 5 mmol/L cyanate before preparing total cell extracts which were then used for β-actin immunoprecipitation. The immunoprecipitates were submitted to acid hydrolysis before HCit quantification by LC-MS/MS (**d**). The data are presented as means ± SEM (n=4) compared using the Mann-Whitney U test (**: p<0.01).

In order to determine which intracellular proteins are most prone to carbamylation, fibroblasts were incubated for a shorter period (5 days) with 5 mmol/L cyanate and carbamylation measured using an anti-HCit immunofluorescence labelling assay (Fig. 3). An intense labelling of the nucleus was observed whereas a more diffused signal was detected in the cytoplasm. These results indicated that cyanate is able to cross the nuclear membrane and thereafter carbamylate nuclear proteins (Fig. 3a). For cytoplasmic proteins, as cytoskeleton proteins exhibit among the longest half-lives among the intracellular proteins [17], a phalloidin-based staining of actin was performed in order to search for colocalization points with HCit labelling (Fig. 3b). Many colocalization points were found (Fig. 3c) demonstrating that actin is one of the preferential targets for carbamylation among the intracellular proteins. To complete these results, β-actin was immunoprecipitated from total cell extracts and HCit was quantified in precipitates in order to determine its carbamylation level (Fig. 3d). Actin was found to be 8.5-fold more carbamylated when fibroblasts were incubated with cyanate in comparison with control conditions (0.23±0.06 *vs* 1.95±0.53 µmol/mol Lys, p<0.05). However, other HCit labelling areas were not colocalized with actin, especially in the perinuclear zone, suggesting that other intracellular proteins are also carbamylated.

### Carbamylation of intracellular proteins alters fibroblast motility but does not induce their senescence

**Figure 4:**
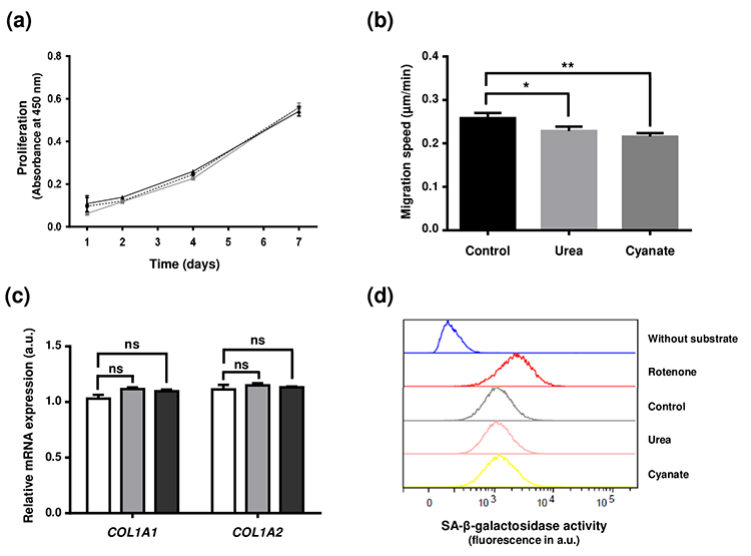
**Effect of intracellular protein carbamylation on cell function and senescence.** Confluent fibroblasts were incubated for 4 weeks at 37°C with DMEM + 0.5% (v/v) FBS without (control conditions) or with 20 mmol/L urea or 0.5 mmol/L cyanate. (**a**) Proliferation: cells were then seeded in 96-well plates at a density of 1,500 cells per well and incubated for 1, 2, 4 and 7 days with DMEM with 10% (v/v) FBS and carbamylating agents. Cell number was evaluated using a WST-1 assay by measuring absorbance at 450 nm. The data presented are means ± SEM (n=6) compared using the Mann-Whitney U test. No significant difference was found between the three conditions (control: dotted line,•; urea: grey line ■; cyanate: black line, ■). (**b**) Cell migration: cells were seeded in 24-well plates at a density of 15,000 cells per well and incubated for 24h at 37°C with DMEM containing 0.5% (v/v) FBS. Pictures of cells were taken every 30 min over the incubation period and each cell (n=58) was followed separately in order to calculate the migration speed. The data are presented as means ± SEM compared using the Mann-Whitney U test (*:p<0.05, **:p<0.01). (**c**) Expression of type I collagen mRNAs: at the end of the 4 weeks-incubation, RNA was isolated from confluent cells and then submitted to RT-qPCR analysis for evaluating the expression of *COL1A1* and *COL1A2* genes. Data represent the relative mRNA expression normalized to *EEF1A1* gene and are expressed as means ± SEM (n=4). The Mann-Whitney U test was used to compare the three conditions: control (open bars), urea (grey bars) and cyanate (black bars). ns: not significant. (**d**) Senescence: cell senescence was determined by measuring the SA-β-galactosidase activity using a C_12_FDG fluorogenic substrate and by detection of senescent cells by flow cytometry. Each plot represents the results of 20,000 events acquired per condition. Incubation of cells with rotenone was used as a positive control of cell senescence whereas a negative control without addition of the fluorogenic substrate was performed.

After demonstrating that carbamylated proteins accumulate intracellularly, this study aimed at evaluating whether these carbamylated proteins could modify various cell functions. Firstly, after long-term incubation (4 weeks) in the presence of urea or cyanate, fibroblasts did not show any morphological change (data not shown). Moreover, viability rates close to 97% were observed in all conditions using the Trypan blue exclusion test (data not shown). A proliferation test was then carried out using cells previously incubated in the presence or absence of carbamylating agents and no significant difference was observed between these treatments (Fig. 4a). As actin was identified as a particular target of carbamylation, the impact of intracellular carbamylation on cell motility was evaluated by performing migration assays (Fig. 4b). The results showed that incubation with carbamylating agents led to a significant decrease of fibroblast migration speed (0.23±0.01 µm/min, -12%, p<0.05 for urea and 0.21±0.01 µm/min, -19%, p<0.01 for cyanate) in comparison with control conditions (0.26±0.01 µm/min). As one of the major functions of fibroblasts is the synthesis of matrix proteins, we determined the levels of expression of *COL1A1* and *COL1A2* genes (*i.e.* genes encoding α_1_ and α_2_ chains of type 1 collagen) after long-term exposure to carbamylating agents. As shown in Fig. 4c, the expression of these two genes was not modified after 4 weeks exposure to urea or cyanate. Finally, as it had been previously demonstrated that carbamylated proteins can induce cell senescence [18], we assessed whether the presence of higher levels of intracellular carbamylated proteins could promote fibroblast senescence. The flow cytometry-based assay using a fluorogenic substrate of the senescence-associated β-galactosidase showed that the cells incubated with the carbamylating agents did not show accelerated senescence when compared with control conditions (Fig. 4d).

### Fibroblasts are able to degrade intracellular carbamylated proteins

**Figure 5:**
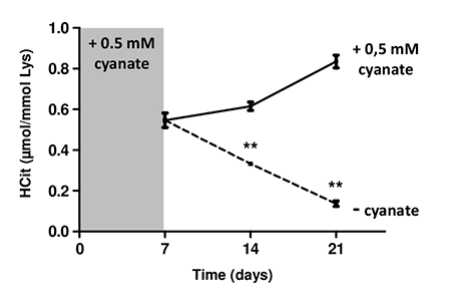
**Intracellular degradation of carbamylated proteins.** Confluent fibroblasts were incubated for 4 weeks at 37°C with DMEM + 0.5% (v/v) FBS and 0.5 mmol/L cyanate in order to induce intracellular protein carbamylation. Cells were then incubated in the same conditions (with 0.5 mM cyanate, solid line) or without cyanate (dotted line) for two additional weeks. HCit content was determined at each time point. The data are presented as means ± SEM (n=6) and the two conditions (with or without cyanate) were compared using the Mann-Whitney U test (**: p<0.01).

In order to investigate the intracellular metabolism of carbamylated proteins, carbamylation was first induced by incubating fibroblasts with 0.5 mmol/L cyanate for 7 days, which resulted in a carbamylation level of 0.55±0.03 µmol/mol Lys (Fig. 5). When the incubation with cyanate was continued for two additional weeks, the carbamylation level increased slightly to 0.61±0.02 µmol/mol Lys at 14 days, and reached 0.83±0.03 µmol/mol Lys at 21 days incubation.

By contrast, when cyanate was removed from the culture medium, a significant (p<0.01) decrease of the carbamylation level was observed. This decrease was equal to -40% and -75% 7 and 14 days after cyanate withdrawal, respectively. HCit values obtained at 21 days were close to those of basal carbamylation conditions (0.14±0.01 µmol/mol Lys). These results demonstrate that fibroblasts were able to degrade intracellular carbamylated proteins.

### 26S proteasome is involved in the degradation of intracellular carbamylated proteins

As 26S proteasome is a key actor in the degradation and the renewal of intracellular proteins [19], we aimed to determine whether this proteasome was able to degrade carbamylated proteins.

**Figure 6:**
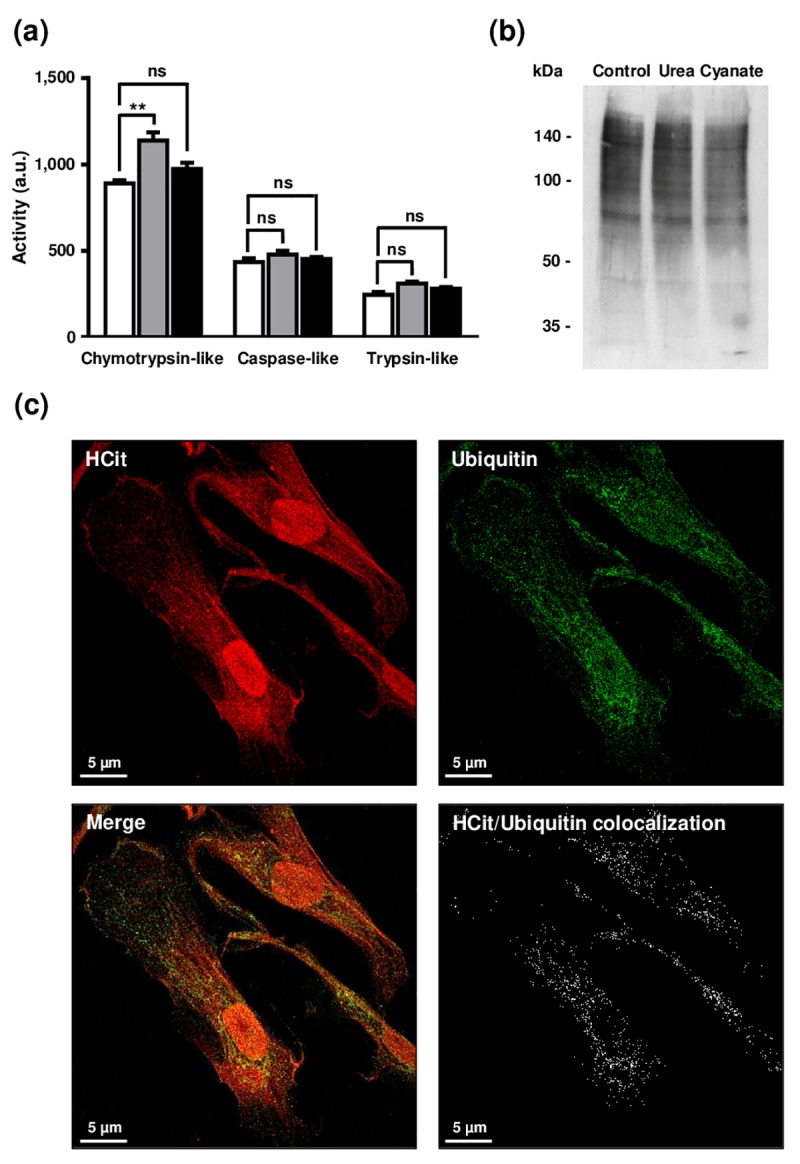
**Impact of carbamylation on proteasome proteolytic activities and on the ubiquitination process.** (**a**) Evaluation of proteasome proteolytic activity after incubation of cells with urea or cyanate: confluent fibroblasts were incubated for 4 weeks at 37°C with DMEM + 0.5% (v/v) FBS without (control conditions, open bars) or with 20 mmol/L urea (grey bars) or 0.5 mmol/L cyanate (black bars). Chymotrypsin-like, caspase-like and trypsin-like activities have been measured in cell extracts using the corresponding Proteasome-Glo™ assays. The data are presented as means ± SEM (n=6) and compared using the Mann-Whitney U test (ns: non significant, **: p<0.01). (**b**) Ubiquitination level of intracellular proteins after incubation of cells with urea or cyanate: confluent fibroblasts were incubated for 4 weeks at 37°C with DMEM + 0.5% (v/v) FBS without (control conditions) or with 20 mmol/L urea or 0.5 mmol/L cyanate, and cell extracts were prepared and submitted to western-blot analysis using an anti-ubiquitin antibody. (**c**) Anti-HCit and anti-ubiquitin immunolabellings were performed using fibroblasts previously seeded (10,000 cells/well) in chambered coverglass system and incubated for 2 days with DMEM containing 0.5% (v/v) FBS and 5 mmol/L cyanate. At the end of incubation, cells were fixed with 4% (v/v) paraformaldehyde and permeabilized with 0.25% (v/v) Triton X-100 before immunolabelling of proteins using both anti-HCit and anti-ubiquitin antibodies. Colocalization points between HCit and ubiquitin labelling were identified using ImageJ software.

Firstly, as it has been reported that carbamylation may lead to a partial or complete loss of activity of different enzymes [20,21], we hypothesized that long-term incubations with urea and cyanate could directly impair proteolytic activities of the proteasome by carbamylation of its catalytic subunits. To test this hypothesis, fibroblasts were incubated for 4 weeks with 20 mmol/L urea or 0.5 mmol/L cyanate, and the proteolytic activities of proteasome (*i.e.* caspase-like, chymotrypsin-like and trypsin-like activities) were then evaluated in cell extracts (Fig. 6a). Chymotrypsin-like activity was increased in cells incubated with urea in comparison with control conditions (1139±48 a.u. *vs* 891±19, p <0.05) but no variation was found after incubation with cyanate. No differences were observed in the other activities (*i.e.* caspase-like and trypsin-like) whatever the conditions, which suggests that a long-term incubation of the cells with these two carbamylating agents does not exert a direct inhibitory effect on 26S proteasome activity.

Secondly, given that the targeting of altered proteins towards the 26S proteasome is dependent on the protein ubiquitination process, we examined whether the increase of protein carbamylation level could interfere with ubiquitination since both reactions occur on lysine residues. For this purpose, cell extracts were prepared following the 4-week incubation of cells with urea or cyanate, and an anti-ubiquitin western-blot analysis performed (Figure 6b). A similar ubiquitination level of proteins was found under all conditions (control, urea and cyanate), which suggests that the carbamylation of intracellular proteins does not interfere with the ubiquitination process. To confirm this result, anti-HCit and anti-ubiquitin immunofluorescence labellings were performed. As seen in Figure 6c, many colocalization points were observed between both labellings suggesting that carbamylation did not greatly interfere with the ubiquitination process, and enough free lysine residues remained for ubiquitin binding.

Finally, to determine the participation of the 26S proteasome in the degradation of intracellular carbamylated proteins, fibroblasts were incubated for three weeks with 0.5 mmol/L cyanate and proteasome inhibitors, namely 10 nmol/L Bortezomib or 500 nmol/L MG-132. Cell viability assessed using the Trypan blue exclusion test was greater than 85% in all conditions (data not shown). In this experiment, results were expressed as µmol HCit per 10^6^ cells because proteasome inhibition also impairs the degradation of non-carbamylated proteins, leading to an increase in lysine content.

**Figure 7:**
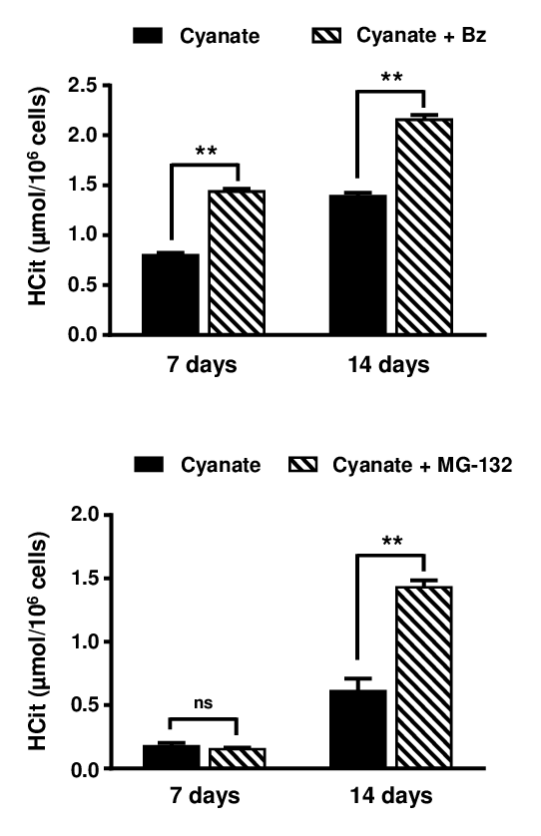
**Role of proteasome in the degradation of carbamylated proteins.** Confluent fibroblasts were incubated for 7 and 14 days at 37°C with DMEM containing 0.5% (v/v) FBS, 0.5 mmol/L sodium cyanate with or without proteasome inhibitors (10 nmol/L Bortezomib (Bz) or 500 nM MG-132). HCit content in total cell extracts was determined by LC-MS/MS. The data are presented as means ± SEM (n=6) and compared using the Mann-Whitney U test (ns: non significant, **: p<0.01).

HCit concentrations were significantly (p<0.01) higher in the presence of the inhibitors at each point of the kinetics for Bortezomib and after only 14 days for MG-132 (Fig. 7). For example, after 14 days of incubation, the carbamylation level was 1.6-fold higher when cells were incubated with Bortezomib than in control conditions (2.16±0.05 *vs* 1.39±0.03 µmol HCit/10^6^ cells, p<0.01) and 2.3-fold higher when cells were incubated with MG-132 (1.43±0.05 *vs* 0.61±0.10 µmol HCit/10^6^ cells, p<0.01). These results demonstrate that the 26S proteasome plays a major role in the degradation of intracellular carbamylated proteins.

## DISCUSSION

In living organisms, proteins are submitted to spontaneous chemical reactions referred to as NEPTMs, which lead to the progressive alteration of their structural and functional properties and thus contribute to their "molecular aging". These modifications which occur at a molecular level may have an impact at the tissue level, especially when they affect extracellular matrix proteins such as collagens. Besides the well-described and extensively studied NEPTMs like oxidation and glycation, the more recently described carbamylation reaction contributes to a similar extent to protein aging [22]. Moreover, while all these NETPMs participate in a cumulative way to protein aging, they may also compete for the same modification sites [23]. We have previously shown that carbamylated type I collagen exhibits altered structural and mechanical properties [24] and that CDPs accumulate in tissues and constitute characteristic features of tissue aging [15]. In addition, carbamylation-modified proteins are able to modulate different cell functions [25,26].

Molecular aging of proteins may also have an impact on cell functions when the intracellular level of modified proteins increases to the point where cellular homeostasis is disrupted. The increase of the proportion of modified proteins inside cells is explained by an increase in their formation and/or by the inability of cells to degrade them, leading to the intracellular accumulation of aggregated proteins [6]. As a consequence, NEPTM-induced modifications of proteostasis can directly participate in cell aging and ultimately be responsible for cell death [5]. Cell aging may also be explained by an increased proportion of modified proteins or enzymes whose efficiency is no longer optimal because of their molecular aging. For example, mitochondrial glutamate dehydrogenase activity was shown to be significantly lower in elderly compared with young rats due to modification of the enzyme by methylglyoxal [27].

The hypothesis of intracellular protein carbamylation was formulated several decades ago when Ramponi *et al.* showed *in vitro* that carbamoyl-phosphate formed during the initial phase of the urea cycle could bind to histones [28]. More recently, the proteomic study of rat renal inner medulla protein extracts identified several intracellular proteins as targets for carbamylation [16]. However, as renal medulla is exposed to very high concentrations of urea (up to 500 mmol/L), the findings of this study were not transposable to other tissue or cell types. Moreover, this qualitative approach did not provide information on the carbamylation level of intracellular proteins nor its evolution over time.

With few data available in the literature concerning the formation of intracellular carbamylated proteins and their role in cell aging, the present study aimed to determine how carbamylated proteins are formed inside cells, their impact on cell functions, and whether cells are able to metabolize these damaged proteins. To this end, we incubated dermal fibroblasts for long periods with carbamylating agents (urea or cyanate). Concentrations of urea and cyanate were chosen to be physiologically relevant: a urea concentration of 20 mM is usually observed in the serum of uremic patients, and the concentration of cyanate was determined taking into account the dissociation rate of urea in aqueous solution [29]. The choice of dermal fibroblasts was made on the basis of our previous work which suggested that skin accumulation of CDPs was not only explained by extracellular matrix proteins carbamylation, but could also involve cell participation [15]. The immunolabelling of human skin sections shown in Fig. 1 confirmed this hypothesis since HCit labelling was clearly found inside fibroblasts. Similar experiments conducted with other cell types (*e.g.* smooth muscle cells) provided similar results (data not shown). Besides, these experiments were carried out on quiescent cells in order to prevent cell multiplication and protein neosynthesis, which could interfere with the observations.

The present study clearly showed that intracellular proteins are carbamylated at a basal level and that this phenomenon is amplified when cells are incubated in the presence of urea or cyanate. It confirms that urea is able to freely diffuse through biological membranes without need for specific urea transporters, such as UT-A or UT-B, which are mainly localized in kidneys [30]. Another evidence for the passive diffusion of urea through cell membranes is the increased formation of carbamylated hemoglobin in uremic patients [31]. In addition, our results suggest that cyanate, like urea, can easily and freely diffuse through cell membranes, and hence membrane and cytosolic proteins exhibit very similar carbamylation levels.

Among the cytoplasmic proteins which may be preferentially targeted by carbamylation, our study focused on cytoskeletal proteins and more particularly on actin, since cytoskeletal proteins are not only among the most abundant proteins in cells but also among those with the longest half-lives [17,32]. Indeed, long-lived proteins like extracellular matrix proteins are known to be preferential targets for NEPTMs because their slow turnover leads to an increased exposure to carbamylating agents. Co-labelling anti-HCit/phalloidin clearly showed that actin under control conditions had a measurable basal level of carbamylation. These results are consistent with the observation of Claxton *et al.* who identified cytoskeletal proteins as preferential protein targets for carbamylation in the renal inner medulla [16]. Such modifications of actin monomers may have consequences on cell mobility since it has been demonstrated that the polymerization of *in vitro* carbamylated actin is impaired [33]. A similar perinuclear localization of carbamylated proteins was observed in our study with fibroblasts. Other cytosolic proteins can be carbamylated as shown by the anti-HCit labelling found in the perinuclear area, as described by others in polynuclear neutrophils [34]. Besides, we observed an intense nuclear labelling which suggests that nuclear proteins are carbamylated. It may be hypothesized that histones are prone to carbamylation as well as to glycation [35]. Indeed, it has been shown that histone molecular aging actively participates in the development of autoimmune diseases [36].

As we observed a significant increase of carbamylated protein amounts at the intracellular level after exposure to urea or cyanate, we then determined whether cell function may be affected. Whereas no effect was observed neither on fibroblast proliferation or the expression of type I collagen genes, cell migration speed was significantly decreased. According to the crucial role of actin in cell motility, this effect may well be explained by the carbamylation of actin which affects its polymerization as described earlier [33]. Moreover, this result is consistent with those described by Sun *et al*., who have recently shown that cyanate and CDPs are responsible for an inhibition of endothelial cell migration [37,38].

Thereafter, we determined whether cells were able to metabolize these damaged proteins. Indeed, highly glycated and/or oxidized proteins form aggregates, insoluble cross-linked pigments (also called lipofuscin), which are stored in cells and cannot be removed. The accumulation of these aggregates usually promotes cellular senescence. By contrast, our results showed that the increased formation of intracellular carbamylated proteins did not lead to premature cellular senescence, suggesting that cells were able to degrade carbamylated proteins in order to prevent their accumulation. Indeed, after the induction of a "carbamylating stress" with cyanate, fibroblasts were able to remove almost all carbamylated proteins within two weeks. Different hypotheses could explain such results, including the direct degradation of damaged proteins by proteolytic systems such as the 26S proteasome or the repair of carbamylated proteins by hypothetical enzymatic mechanisms. We chose to focus on the role of the proteasome in this phenomenon. Experiments carried out in the presence of specific inhibitors (*i.e.* Bortezomib and MG-132) clearly showed that the proteasome participates in the removal of intracellular carbamylated proteins. However, our results do not allow us to determine whether the carbamylated proteins are degraded at the same rate as the native proteins, knowing that a slower degradation could contribute to the intracellular accumulation of carbamylated proteins. Further experiments are needed to address this question.

The fact that cells are able to degrade carbamylated proteins is an interesting feature which differentiates carbamylation from oxidation- and glycation-induced damages, since highly oxidized and glycated proteins, which exhibit elevated rates of intra- and intermolecular cross-linking, are resistant to degradation by the proteasome [39].

We also hypothesized that carbamylation could interfere with the ubiquitination process, as carbamylated lysine residues are no longer available for ubiquitin binding. However, this effect appears to be negligible since ubiquitination levels of intracellular proteins were similar even when cells had been incubated with carbamylating agents. Moreover, anti-HCit and anti-ubiquitin immunolabelling showed that carbamylated proteins may be also ubiquitinylated. Finally, since it has been shown that enzymatic activities of the proteasome catalytic subunits were altered by glycation [40], we evaluated the direct effect of carbamylation on chymotrypsin-like, caspase-like and trypsin-like activities. No significant carbamylation-induced decrease in proteasome enzymatic activities was observed.

In conclusion, our results show that intracellular proteins may be carbamylated and that cells are able to degrade these modified proteins, mainly through the ubiquitin-proteasome system, contrary to highly oxidized and glycated proteins. Excepting a decrease in cell migration, no modification of other cell functions was observed, nor was cell viability affected. Thus, the ability of cells to degrade intracellular carbamylated proteins *via* the ubiquitin-proteasome system seems to limit the potential toxic effect of CDPs at the intracellular level, in contrast with the effects observed in the extracellular space.

## MATERIALS AND METHODS

### Reagents

Dulbecco’s Modified Eagle’s Medium (DMEM), Penicillin and Streptomycin (P/S, PenStrep™), Bovine Serum Albumin (BSA), Phosphate Buffered Saline (PBS), Trypsin-EDTA (15400054), Alexa Fluor™488 donkey anti-rabbit IgG, Alexafluor-568 goat anti-rabbit IgG and ActinRed™ 555 ReadyProbes™ reagent (R37112) were purchased from Thermo Fisher Scientific (Villebon-sur-Yvette, France). Triton X-100 (T9284), EDTA (E-5134), urea (EU0014-B), SIGMA*FAST*™ proteinase inhibitor coktail (s8820) and formaldehyde solution (F8775) were from Sigma-Aldrich (St Louis, MO, USA), and sodium cyanate (NaCNO, A12413) from VWR (Radnor, PA, USA), and Bortezomib and MG-132 from Selleckchem (Souffel Weyersheim, France). Rabbit polyclonal antibody anti-HCit was produced by Covalab (Villeurbanne, France) and purified in the laboratory [15] whereas anti-ubiquitin monoclonal antibody (sc-8017) was from Santa-Cruz (Dallas, TX, USA). Control rabbit polyclonal IgG (ab27478), anti-β-actin monoclonal antibody (ab8226) and protein A/G sepharose beads were purchased from Abcam (Cambridge, UK). Fetal Bovine Serum (FBS) was from Dutscher (Issy-les-Moulineaux, France), Trypan blue from Sciencetec (Villebon-sur-Yvette, France) and DAPI-Fluoromount-G™ from SouthernBiotech (Birmingham, AL, USA). The Proteasome-Glo™ Chymotrypsin-Like, Trypsine-Like and Caspase-Like Cell-Based Assays were from Promega (Madison, WI, USA). Bradford protein assay was from Bio-Rad (Marnes-la-Coquette, France).

### Cell culture

Human dermal fibroblasts were purchased from PromoCell (batch number: 0083002.2, Heidelberg, Germany). Cells were cultured in DMEM containing glucose 1g/L with 0.5% (v/v) FBS and 1% (v/v) P/S in 75 cm^2^ or 150 cm^2^ culture flasks (Corning, Life Sciences, Durham, NC, USA) in a humid atmosphere (5% CO2, 95% air). Different carbamylating agents were added in the culture medium: urea at 20 mmol/L or sodium cyanate (NaCNO) at 0.5 mmol/L or 5 mmol/L. The lack of toxicity was checked by using different assays (trypan blue and WST-1 assays) and by assessing the absence of morphological changes of cells (data not shown). No cellular toxicity was observed up to 80 mM urea and 10 mM cyanate for the corresponding incubation times. For the experiments aiming to determining the role of the proteasome in the degradation of carbamylated proteins, cells were incubated with 10 nmol/L Bortezomib or 500 nmol/L MG-132.

### Cell proliferation

Fibroblasts previously incubated for 4 weeks in DMEM containing 0.5% (v/v) FBS and carbamylating agents (0.5 mmol/L cyanate or 20 mmol/L urea) were seeded in 96-well plates at a density of 1,500 cells per well. They were then incubated for 1, 2, 4 and 7 days with DMEM with 10% (v/v) FBS and carbamylating agents. At the end of incubation, the number of cells was evaluated using a WST-1 assay (Roche, Switzerland) by measuring absorbance at 450 nm.

### Cell migration

Fibroblasts previously incubated for 4 weeks in DMEM containing 0.5% (v/v) FBS and carbamylating agents (0.5 mmol/L cyanate or 20 mmol/L urea) were seeded in 24-well plates at a density of 15,000 cells per well. Cell migration was analyzed by time-lapse videomicroscopy using an inverted microscope Axioobserver ZEISS (Carl Zeiss SAS, Germany) equipped with a small transparent environmental chamber Climabox (Zeiss), at 37˚C in an humidified atmosphere containing 5% (v/v) CO_2_. The microscope was driven by the Metamorph software (Roper Scientific, Evry, France) and images were recorded with a charge-coupled device camera CoolsnapHG2 bining 2 (Roper Scientific). Pictures of cells were taken every 20 min over a 24h period and cell migration speed was quantified (μm/min) for each individual non-mitotic cell (n=16) using an interactive tracking method (Fiji-Manual Tracking software).

### RNA isolation and RT-qPCR analysis

Total RNAs were isolated from fibroblasts previously incubated for 4 weeks in DMEM containing 0.5% (v/v) FBS and carbamylating agents (0.5 mmol/L cyanate or 20 mmol/L urea) using the RNeasy Plus Mini Kit 50 (QIAGEN) according manufacturer's instructions. Reverse transcription (RT) was then performed from 1 µg RNA using the Maxima First Strand cDNA Synthesis Kit for RT-qPCR (ThermoFisher). Quantitative polymerase chain reaction (qPCR) was thereafter conducted in 20 μL reaction mixture using Maxima SYBR Green/ROX qPCR (ThermoFisher) and the following primers for *COL1A1* (Forward: 5’AAG ACG AAG ACA TCC CAC CA 3’, reverse: 5’ GCA GTT CTT GGT CTC GTC AC 3’), *COL1A2* (Forward: 5’ TTT CCC TGG AAC TCC TGG AC 3’, reverse: 5’ AGG TTC ACC CTT CAG ACC AG 3’) and *EEF1A1* (Forward: 5’ TAT CCA CCT TTG GGT CGC TT 3’, reverse: 5’ ACC GTT CTT CCA CCA CTG AT 3’). The relative expression of the different genes was calculated by the ΔΔCt method. The Ct of any gene of interest was normalized to the Ct of the reference gene (*EEF1A1*).

### Cell senescence

Cell senescence was determined by measuring the senescence-associated (SA)-β-galactosidase activity. This method is based on the use of a fluorogenic substrate (C_12_FDG: 5-dodecamoylaminofluorescein di-β-galactopyranoside) of SA-β-galactosidase and the detection of senescent cells by flow cytometry. For this purpose, fibroblasts were incubated for 4 weeks with carbamylated agents as described above. A positive control of senescence state was performed by incubating control fibroblasts for 72h with 0.1 µM rotenone.

To induce lysosomal alkalinisation, cells were incubated for one hour with 100 nM bafilomycin in fresh culture medium at 37°C, before the addition of the C_12_FDG substrate (final concentration: 33 µmol/L) for one hour. Culture medium was removed and cell monolayers were washed twice for 30 s with a PBS solution at room temperature. After incubation with trypsin-EDTA, cells were resuspended in 200 µL of ice-cold PBS solution and analyzed by flow cytometry (LSR Fortessa instrument, BD Biosciences, Franklin Lakes, NY, USA). Results of 20,000 events acquired per condition were represented in a plot where the x-axis indicates C_12_-fluorescein fluorescence intensity in a log scale. The β-galactosidase activity was estimated using the median fluorescence intensity.

### Total protein extraction

Total protein extraction was performed from fibroblasts cultured for 4 weeks in DMEM containing 0.5% (v/v) FBS and 1% (v/v) P/S, with or without 20 mmol/L urea or 0.5 mmol/L cyanate. At the end of the incubation in a 150 cm^2^ flask, medium was removed and cells were washed with 10 mL of PBS. Cells were collected after 10 min incubation at 37°C with 10 mL of 0.05% (m/v) trypsin and 0.53 mmol/L EDTA in PBS. Cell viability was measured using Trypan blue exclusion test. After centrifugation (300g, 5 min, 20°C) the cell pellet was lysed by adding 600µL distilled water and stored at -80°C until LC-MS/MS quantification of HCit.

### Membrane and cytoplasmic protein extraction

After fibroblasts had been cultured in the same conditions as described above, membrane and cytoplasmic proteins have been extracted using the mem-PER™ Plus Kit (ref. number 89845, ThermoScientific, Rockfrod, IL, USA). Briefly, after the 4-weeks incubation at 37°C, cells were washed with 10 mL PBS and collected after 10 min incubation at 37°C in 10 mL of PBS containing 1 mmol/L EDTA. Cells were then washed with PBS and centrifuged (300g, 5 min, 20°C). The supernatant was discarded and the cell pellet was mixed with 650 µL of permeabilization buffer, vortexed and incubated for 10 min at 4°C under constant agitation. After centrifugation (16,000 g, 15 min, 4°C), the supernatant, which corresponds to the cytosolic fraction, was kept for further analyses and the pellet was homogenized with 650 µL of solubilization buffer and incubated for 30 min at 4°C under constant agitation. After centrifugation (16,000 g, 15 min, 4°C), the supernatant (corresponding to the membrane fraction) was transferred into a new microtube. In order to remove solvents contained in permeabilization and solubilization buffers, the two fractions were dialyzed for 24 h against distilled water using MIDI GeBaFlex-tubes (cutoff 8 kDa, Gene Bio-Application, Kfar-Hanagid, Israel).

### HCit and lysine quantification

Samples were hydrolyzed by 6 mmol/L (final concentration) hydrochloric acid for 18h at 110°C. Hydrolysates were evaporated to dryness under a nitrogen stream. After addition of 1 mL of distilled water, a new evaporation was performed in order to remove residual acidity. Dried samples were resuspended in 100 µL ammonium formate 125 mmol/L containing 1 µmol/L of d_7_-citrulline and 65 µmol/L of d_8_-lysine used as internals standards (IS), and filtered using Uptidisc PTFE Filters (0.45 µm, Phenomenex, Le Pecq, France). For HCit quantification, samples were 5-fold diluted in 125 mmol/L ammonium formate containing the IS. For lysine quantification, samples were 10-fold diluted in 125 mmol/L ammonium formate containing the IS and then 5-fold in 5 mmol/L ammonium formate (pH 2.9) containing 65 µmol/L d_8_-lysine. HCit and lysine were assayed in diluted hydrolysates by LC-MS/MS (Shimadzu, API 4000; ABSciex) as previously described [15,41]. Results were expressed as ratios of HCit to lysine.

### HCit, ubiquitin and actin labelling

Anti-HCit immunofluorescence labelling was performed using fibroblasts seeded (10,000 cells per well) in a chambered cover glass system (Lab-Tek, ThermoFisher Scientific, Rochester, NY,USA) and incubated for 5 days in DMEM containing 0.5% (v/v) FBS, 1% (v/v) P/S and 5 mmol/L cyanate. At the end of incubation, the medium was removed and the cells were washed with PBS and fixed with 4% (m/v) paraformaldehyde for 10 min at room temperature. Fixed cells were washed three times for 5 min with PBS and permeabilized with 0.25% (v/v) Triton X-100 in PBS for 10 min at room temperature. Cells were washed three times with PBS and nonspecific sites were saturated by incubation with 3% (m/v) BSA in PBS for 30 min. Cells were then incubated for 12h at 4°C with anti-HCit polyclonal antibody or the corresponding control IgG, both diluted 120-fold in PBS containing 1% (m/v) BSA. After three washes with PBS, the secondary antibody (AlexaFluor™ 488 donkey anti-rabbit 100-fold diluted in BSA 1% (m/v) in PBS) was added for one hour at room temperature. After another set of three washes with PBS, cells were incubated 30 min at room temperature in darkness with ActinRed 555 ReadyProbes™ reagent diluted 50-fold in PBS containing 1% (m/v) BSA. Finally, cells were washed 3 times in PBS, and were incubated in DAPI-Fluoromount-G™ at 4°C in darkness. Cells were observed with a LSM 710-NLO confocal microscope coupled to laser Chameleon femtosecond Titanium-Sapphire (Platform of Cellular and Tissular Imagery, University of Reims Champagne-Ardenne). Image treatment was performed with the public open source software ImageJ (Bethesda, MD, USA). Co-localization points correspond to a 90% rate of pixel overlay between HCit and actin labelling in a single cell z-cross-section.

For the simultaneous anti-HCit and anti-ubiquitin immunofluorescence labelling, fibroblasts were seeded (10,000 cells per well) in Lab-Tek coverglass system, and incubated for 5 days in DMEM containing 0.5% (v/v) FBS, 1% (v/v) P/S and 5 mmol/L cyanate. At the end of incubation, washes, fixation of cells and incubation with antibodies were performed as described in the above paragraph. The anti-ubiquitin monoclonal antibody used for this experiment was directly coupled with Alexafluor 488 and diluted 120-fold (v/v) in PBS containing 1% (m/v) BSA. The secondary antibody raised against anti-HCit antibody (Alexafluor-568 goat anti-rabbit IgG) was 100-fold (v/v) diluted in BSA 1% (m/v) in PBS. Cells were then observed as described above with a confocal microscope and image treatment performed with ImageJ software. Co-localization points correspond to a 90% rate of pixel overlay between HCit and ubiquitin labelling in a single cell z-cross-section.

### Immunohistolabelling of HCit in human skin

HCit immunolabelling was realized in several skin samples obtained from humans after protocol of body donation to science following death according to French regulations. The skin sample from a 77-year old man used for this article is an example showing a typical labelling. The samples were cleaned of adipose tissue and embedded in a cryomatrix (Shandon, Thermo Scientific) then frozen in liquid nitrogen before storage at -80°C. From cryomatrix block, 6-µm thick sections were performed with a microtome (MICROM Cryo-Star HM 560). After rehydration and washes with PBS, sections were incubated with anti-HCit antibody (rabbit polyclonal antibody, dilution 1:40, Covalab) overnight at 4°C. After serial washes with PBS, sections were incubated with anti-rabbit IgG antibody labelled with Alexa Fluor 488 (dilution 1:100, Invitrogen) for 30 min at room temperature. The sections were counterstained with DAPI (Dapi-Fluoromount G, Clinisciences) and hematoxylin-eosin staining.

### β-actin immunoprecipitation

Confluent fibroblasts were incubated for 5 days in DMEM containing 0.5% (v/v) FBS with or without 5 mmol/L cyanate in a 150 cm^2^ flask. At the end of the incubation, the medium was removed, the cells were washed with 10 mL of PBS and collected after 10 min incubation at 37°C with 10 mL of 0.05% (m/v) trypsin and 0.53 mmol/L EDTA in PBS. After centrifugation (300g, 5 min, 20°C), the cell pellet was lysed by adding 500 µL lysis buffer containing 75 mM Tris, 2 mM EDTA, 12 mM MgCl_2_, proteinase inhibitor cocktail, 10 mM NaF, 2 mM Na_3_VO_4_, 1% CHAPS, pH 7.5. After sonication, lysates were then centrifuged (20,000g, 45 min, 4°C) and protein content in the supernatant was determined using Bradford protein assay. In parallel, protein A/G sepharose beads were washed three times with lysis buffer and saturated with PBS containing 2% (m/v) BSA overnight at 4°C. One mg of proteins was mixed with 50 µg anti-β-actin antibody and 100 µL of saturated beads. After a 18h-incubation at 4°C, the beads were washed three times with lysis buffer and were then submitted to acid hydrolysis prior to HCit quantification by LC-MS/MS (acid hydrolysis and HCit quantification are described above).

### Assessment of ubiquitination level of intracellular proteins by western-blot analysis

After a 4-week incubation of fibroblasts with carbamylating agents, cells were washed with PBS and lyzed using a specific buffer (50 mM Tris-HCl pH 8.0; 150 mM NaCl; 1% Igepal CA-630; 0.5% (m/v) sodium deoxycholate; 0.1% sodium dodecyl sulphate) containing 1% (m/v) protease inhibitors cocktail (Hall Protease Inhibitor Cocktail, Thermo Scientific, Rockford, IL, USA). Protein content in cell lysates was determined using Bradford assay (Protein Assay Dye Reagent Concentrate, Bio-Rad, Hercules, CA, USA). Ten micrograms of proteins were then separated by 8% (m/v) SDS-PAGE and transferred to a PVDF membrane. The membrane was blocked with Tris-buffered saline (Tris HCl 20mM pH 7.5, NaCl 150mM) containing 0.1% Tween (v/v) (TBS-T) and 5% (w/v) fat-free dehydrated milk, at room temperature for 1 hour. After three washes with TBS-T, the membrane was incubated overnight at 4°C with an anti-ubiquitin antibody directly coupled to horseradish peroxidase (Santa Cruz, Dallas, USA, used at 1/120 dilution in TBS-T containing 1% (m/v) dry milk). Finally, detection was performed using an ECL Prime Western Blotting Detection kit (GE Healthcare) according to the manufacturer’s protocol.

### Proteasome activity assays

Chymotrypsin-like, trypsin-like and caspase-like proteasome activities were measured separately using the Proteasome-Glo™ Cell-Based Assays. Each reagent contained a specific luminogenic substrate, which was recognized and cleaved by the proteasome, releasing aminoluciferin and allowing the luciferase reaction to produce a luminescent signal, proportional to the enzymatic activity.

Assays were performed in fibroblasts cultured for 4 weeks in DMEM containing 0.5% (v/v) FBS and 1% (v/v) P/S, with or without 20 mmol/L urea or 0.5 mmol/L cyanate. Ten thousand cells/well were incubated for 2 hours in a 96-well plate in a volume of 100 µL. Then, 100 µL of reagent Proteasome-Glo™ was added and luminescence measured using FLUOstar Omega (BMG Labtech, Champigny-sur-Marne, France) after 5 minutes of incubation at room temperature and expressed as arbitrary units (a.u.).

### Statistical analysis

All statistical analyses were performed using GraphPad 6.0 Prism Software. Results were expressed as means ± standard errors of the mean (SEM). Data were compared using the Mann Whitney non-parametric U-test and were considered significantly different when *p*<0.05.
